# Dietary Patterns Are Associated with the Gut Microbiome and Metabolic Syndrome in Mexican Postmenopausal Women

**DOI:** 10.3390/nu15224704

**Published:** 2023-11-07

**Authors:** Priscilla López-Montoya, Berenice Rivera-Paredez, Berenice Palacios-González, Sofia Morán-Ramos, Blanca E. López-Contreras, Samuel Canizales-Quinteros, Jorge Salmerón, Rafael Velázquez-Cruz

**Affiliations:** 1 Laboratorio de Genómica del Metabolismo Óseo, Instituto Nacional de Medicina Genómica (INMEGEN), Mexico City 14610, Mexico; priscilla_lopez92@comunidad.unam.mx; 2 Centro de Investigación en Políticas, Población y Salud de la Facultad de Medicina, Universidad Nacional Autónoma de México (UNAM), Mexico City 04510, Mexico; bereriveraparedez7@gmail.com (B.R.-P.);; 3 Laboratorio de Envejecimiento Saludable, Centro de Investigación Sobre Envejecimiento, Instituto Nacional de Medicina Genómica (INMEGEN), Mexico City 14330, Mexico; bpalacios@inmegen.gob.mx; 4 Departamento de Alimentos y Biotecnología, Facultad de Química, Universidad Nacional Autónoma de México (UNAM), Mexico City 04510, Mexico; sofi_moran@yahoo.com.mx; 5 Unidad de Genómica de Poblaciones Aplicada a la Salud, Facultad de Química, Universidad Nacional Autónoma de México (UNAM)/Instituto Nacional de Medicina Genómica (INMEGEN), Mexico City 14610, Mexico; blopez@inmegen.gob.mx (B.E.L.-C.); cani@unam.mx (S.C.-Q.)

**Keywords:** metabolic syndrome, postmenopausal women, gut microbiota, dietary patterns, macronutrients

## Abstract

Postmenopausal women are at an increased risk of developing metabolic syndrome (MetS) due to hormonal changes and lifestyle factors. Gut microbiota (GM) have been linked to the development of MetS, and they are influenced by dietary habits. However, the interactions between dietary patterns (DP) and the GM of postmenopausal women, as well as their influence on MetS, still need to be understood. The present study evaluated the DP and microbiota composition of postmenopausal Mexican women with MetS and those in a control group. Diet was assessed using a food frequency questionnaire, and the GM were profiled using 16S rRNA gene sequencing. Greater adherence to a “healthy” DP was significantly associated with lower values of MetS risk factors. GM diversity was diminished in women with MetS, and it was negatively influenced by an “unhealthy” DP. Moreover, a higher intake of fats and proteins, as well as lower amounts of carbohydrates, showed a reduction in some of the short-chain fatty acid-producing genera in women with MetS, as well as increases in some harmful bacteria. Furthermore, *Roseburia* abundance was positively associated with dietary fat and waist circumference, which may explain 7.5% of the relationship between this macronutrient and MetS risk factors. These findings suggest that GM and diet interactions are important in the development of MetS in postmenopausal Mexican women.

## 1. Introduction

Metabolic syndrome (MetS) is a highly prevalent, multifaceted condition characterized by several metabolic disorders, including impaired fasting glucose, hypertriglyceridemia, low high-density lipoprotein cholesterol (HDL-C), hypertension, and abdominal obesity [1]. According to the Adult Treatment Panel III (ATP III) criteria, the prevalence of MetS varies between 12% and 37% in the adult population across the World Health Organization (WHO) regions, and it also continues to increase worldwide [2]. The National Health and Nutrition Survey (ENSANUT) survey of 2018 reported that approximately 5 out of 10 Mexican adults have MetS (54.2%), which is one of the higher prevalences worldwide [3]. Moreover, prevalence rates were higher in Mexican women (59.0%) than in men (53.2%), and this was related to the aging of the population [3].

The etiology of MetS encompasses several complex interactions between genetic, epigenetic, environmental, and lifestyle factors [4]. The postmenopausal stage has been associated with a higher odds (OR 2.75) of developing MetS [5]. Regarding this, aging and hormonal changes during the menopausal period are probably the principal causes of the accumulation of MetS risk factors, and these mainly result in the redistribution of adipose tissue in the abdominal region [6]. Also, high caloric intake is an important trigger of visceral adiposity and, consequently, the activation of MetS-related pathways [7,8]. 

Furthermore, accumulating evidence suggests that the complex interactions among dietary habits, sex hormones, and gut microbiota during the postmenopausal stage play a pivotal role in the development of risk factors and the progression of MetS [9,10,11]. Dietary habits, particularly diet composition and quality, profoundly influence the gut microbiota [12]. For example, a Western-style diet characterized by high saturated fats, refined sugars, and low fiber intake has been associated with dysbiosis and an increased risk of MetS [13]. Conversely, a diverse and fiber-rich diet, such as the Mediterranean diet, promotes the growth of beneficial bacteria and enhances microbial diversity, thereby exerting protective effects against MetS [13,14]. Moreover, the influence of an inadequate diet in conjunction with the depletion of beneficial bacteria due to the loss of conjugated sex steroid substrates during menopause could be related to adverse metabolic risk [9,11]. Thus, this study aimed to evaluate the interactions between dietary patterns and gut microbiota in Mexican postmenopausal women, as well as their impact on MetS.

## 2. Materials and Methods

### 2.1. Study Population

The Health Workers Cohort Study (HWCS) is a prospective study with three follow-ups, and it was designed to explore the role of genetic and environmental factors in chronic diseases. The participants were employees of the Mexican Institute of Social Security (IMSS as per its Spanish acronym), and their relatives resided in the urban areas of central Mexico. Details of the study design and methods have been published previously [15]. In the third recruitment (2016–2019), stool samples were collected—using stool collection kits and an *n* = 1050—from a subsample of the participants at home, as was previously described [16]. 

A total of 116 unrelated postmenopausal women were selected, and postmenopausal status was based on an affirmative response to the medical reproductive history question “Have your menstrual periods stopped permanently?”. The inclusion criteria were 12 consecutive months without menstruation and the presence of 5 or no metabolic syndrome risk factors [17]. Older women were considered to be ≥65 years old. Women younger than 45 years and those who reported taking (i) estrogen therapy, (ii) hormonal birth control, or (iii) antibiotics three months before recruitment were excluded. Sociodemographic and lifestyle characteristics, as well as detailed medical history, were obtained using a self-administered questionnaire. Leisure-time physical activity was evaluated using a validated questionnaire on physical activity [18]. The project was approved by the IMSS (no. 12CEI 09 006 14, 17 May 2016) and the National Institute of Genomic Medicine (314-CEI 2018/13, 6 March 2018, and CEI 2023/25, 19 June 2023); moreover, the project followed the guidelines detailed in the Declaration of Helsinki, and all the participants provided written informed consent.

### 2.2. Metabolic Syndrome

Metabolic syndrome (MetS) was classified according to the criteria established by the National Cholesterol Education Program Adult Treatment Program III (NCEP-ATP III), and it was defined as the presence of five of the following risk indicators: (i) elevated fasting glucose (≥100 mg/dL), (ii) elevated triglycerides (TG ≥ 150 mg/dL), (iii) low high-density lipoprotein cholesterol (HDL-C < 50 mg/dL), (iv) elevated blood pressure (systolic [SBP] > 130 mmHg or diastolic [DBP] > 85 mmHg), and (v) abdominal obesity (waist circumference [WC] ≥ 108 cm) [17].

### 2.3. Clinical, Anthropometric, and Biochemical Evaluation

Waist circumference was assessed using a steel measuring tape positioned at the highest point of the iliac crest after a normal exhale, which was then rounded to the nearest 0.1 cm. The participants’ weight was measured using a calibrated electronic TANITA scale while wearing minimal clothing. Height was measured using a standard stadiometer. The body mass index (BMI) was calculated as the ratio of weight (kg) to height squared (in square meters). Blood pressure was assessed using an electronic digital monitor (OMROM HEM-907). The participants were seated with their right arm positioned at heart level. Trained nurses measured anthropometric criteria and blood pressure using standardized procedures, thus ensuring reproducibility (evaluated with a concordance coefficient of 0.83–0.90). Fasting venous blood samples were collected for analysis. Glucose levels were measured using the oxidase glucose method. Triglycerides were determined using a colorimetric method that followed a enzymatic hydrolysis with lipases, whereas the HDL-C was assessed by eliminating chylomicrons and employing a subsequent catalase. Each biomedical assay was processed using a Selectra XL instrument (Randox Laboratories Ltd., Antrim, UK). All parameters were measured at the IMSS laboratory in Cuernavaca, Morelos, following standardized procedures in accordance with the guidelines of the International Federation of Clinical Chemistry and Laboratory Medicine [19].

### 2.4. Dietary Intake Assessment

The habitual diet was assessed using a semi-quantitative food frequency questionnaire (FFQ) that had been previously validated in the Mexican population [20]. The questionnaire included the frequency of consumption of 116 food items during the previous 12 months. Data were converted into daily energy and nutrient intake using the Evaluation System of Nutritional Habits and Nutrient Intake software (v3.0) [21]. Dietary patterns were derived from factor analysis of 28 groups, which were categorized as previously described [22]. To enhance interpretability and ensure uncorrelated factors, the factors were subjected to orthogonal rotation. This was achieved specifically using the varimax method, which is a commonly employed technique in dietary pattern analysis. After evaluating the eigenvalues, screen plot test results, and interpretability, we retained three factors with values of >1.5. To determine the contribution of specific food groups to each pattern, we considered food groups with absolute factor loadings of ≥0.3 as significant contributors. A higher factor score for a particular pattern indicated a higher intake of foods associated with that pattern, while a lower score indicated a lower intake of those foods. Additionally, the dietary patterns were adjusted for energy intake using the residual method. The adjustment for energy intake was a crucial step in our analysis as it allowed us to examine the dietary patterns while accounting for variations in the total energy consumption among individuals. By adjusting for energy intake using the residual method, we were better able to evaluate the dietary patterns independently of the influence of total energy intake. This ensured that our analysis focused on the composition of the diet rather than the total amount of food consumed [23]. The study groups were also divided into categories that were defined by tertiles according to their dietary pattern scores. Women with no diet information or outlier energy intake values over 3.86 standard deviations (outside the range of 500–6500 kcal) were excluded from the diet analyses [24]. 

### 2.5. Stool Sampling and DNA Extraction

Fecal samples were collected in a sterile container, cold-chain-transported, and stored at −80 °C in aliquots of 200 mg until required for processing. DNA was extracted using a QIAamp^®^ DNA Stool Minikit or Power Fecal Pro Kit (QIAGEN, Hilden, Germany), and this was conducted following the manufacturer’s instructions, as well as by adding a previous step of mechanical sample lysis with a FastPrep device. DNA concentration and purity were determined using spectrophotometry (Nanodrop 2000c; Thermo Scientific, Wilmington, DE, USA).

### 2.6. 16S rRNA Sequencing

DNA samples were sequenced using the “Earth Microbiome Project” primers 515F and 806R for the 16S rRNA gene V4 hypervariable region, as has been previously described [25]. Briefly, the first PCR was performed on 100 ng of DNA, and the products were purified using AMPure XP beads (Beckman Coulter, Indianapolis, IN, USA). Equimolar ratios of the individual amplicons were pooled, and a second PCR was run to incorporate dual indices and Illumina sequencing adapters. The resulting pooled libraries were also purified with AMPure XP beads Amplicon; their sizes and concentrations were assessed using an Agilent TapeStation 4200 (Agilent Technologies, Santa Clara, CA, USA) and a Qubit 3.0 fluorometer (Invitrogen, Waltham, MA, USA), respectively. DNA libraries were sequenced at the Sequencing Unit of the National Institute of Genomic Medicine (INMEGEN as per its Spanish acronym) using an Illumina MiSeq 2 × 250 platform (Illumina, San Diego, CA, USA).

### 2.7. Sequence Data Processing

Paired-end raw reads were processed using the QIIME2 pipeline [26]. Demultiplexed reads, both forward and reverse, were trimmed at 30 bp and truncated at 220 bp. The reads were denoised using the DADA2 plugin to resolve the amplicon sequence variants (ASV’s), and chimeric sequences were removed using the “consensus” method [27]. After resolution, the SILVA v138-99 reference database was used to assign taxonomy to each representative ASV sequence [28]. The ASV’s were aligned with the MAFFT algorithm, and a phylogeny tree was built with the FastTree algorithm [29,30]. Mitochondrial and plastid ASV’s were filtered out, and samples were standardized by rarefaction at an 18,630 high-quality read depth with a total of 2,161,080 reads. Women with <10,000 sequence reads per sample were excluded from the study.

### 2.8. Bioinformatic Analysis

The data were exported to the R environment (v4.2.3) for further analysis with the phyloseq package (v1.42.0) [31]. Alpha diversity was calculated using observed ASV, Chao1, Shannon, and Simpson indices. Beta diversity was estimated using weighted and unweighted UniFrac distances. A permutational multivariate analysis of variance (PERMANOVA) was carried out with the vegan package (v2.6.4), whereby 10,000 permutations were applied to test the differences in the beta diversity between study groups [32]. Differential abundance analysis at all taxonomic levels were performed with the linear discriminant analysis effect size (LEfSe v1.0), which was achieved by considering an LDA score of >2 and a *p* < 0.05 to determine what was statistically significant [33]. Covariate effects on microbiota abundance association with MetS risk indicators and diet intake were assessed using multivariate linear models (MaAsLin2 v1.12.0) with default parameters [34]. The mediation effect of microbiota genera on diet intake and MetS risk indicators association was evaluated as follows: (i) diet significantly predicts MetS risk indicators (Y = b_0_ + b_1_X + e_1_), (ii) diet significantly predicts microbiota genera abundance (M = b_0_ + b_2_X + e_2_), (iii) microbiota genera significantly predict MetS risk indicators (Y = b_0_ + b_3_M + b_4_X + e_3_) while adjusting diet intake, and (iv) the relationship between diet and MetS risk indicators when weakened controls for microbiota genera abundance. The average causal mediation effect (b_2_ × b_3_), average direct effect (b_4_), and total effect (b_1_) were estimated using the mediation package (v4.5.0).

### 2.9. Statistical Analysis

All statistical analyses were performed using R software (v4.2.3). Continuous non-normally distributed variables were compared using the Mann–Whitney U test, whereas the *X*^2^ test was used to compare the categorical variables. Diversity indices were compared using a two-tailed Student’s *t*-test. Spearman’s correlation was tested using the psych package (v2.3.3). Linear models were assessed using the lme4 package (v1.1.32), adjusted for age, T2D diagnosis, as well as glucose, lipid, and blood pressure-lowering treatment. Statistical significance was defined at *p* < 0.05, and the *p*-values were corrected for multiple testing using the Benjamini–Hochberg method [35]. All plots were created using the ggplot2 package (v3.4.1).

## 3. Results

### 3.1. Description of Study Population

Women with MetS were significantly older and had a higher body mass index (BMI) than those in the control group. Approximately half of the MetS group reported a use of glucose-, lipid, and blood pressure-lowering drugs, as well as being diagnosed with type 2 diabetes (T2D). The differences in the total energy intake and physical activity between the groups were not significant (Table 1).

### 3.2. Dietary Patterns in MetS and Control Groups

Food intake data for the 28 predefined food groups were entered into the factor analysis procedure with the principal components. The first factor, which accounted for 8.5% of the variance, was characterized by a high consumption of fresh vegetables, fresh fruit, fruit juices, fish and seafood, oils and nuts, and alcohol, as well as a lower consumption of corn tortillas, refined grains, desserts, butter, and other sweetened beverages; this type of diet was labeled a “healthy” dietary pattern. Factor two, which we labeled as “unhealthy”, was characterized by high consumption of pastry, sweets and sugar, sodas, low-energy drinks, alcohol, and tea and caffeine, as well as lower consumption of fresh vegetables, legumes, and other sweetened beverages; this second group accounted for 8.0% of the variance. Lastly, the third factor, defined as “protein”, accounted for 7.4% of the total variance and was characterized by high consumption of potatoes, eggs, poultry, red meat, processed meat, fish and seafood, butter, and water, as well as lower consumption of pastry, desserts, and other sweetened beverages. Together, these three dietary patterns explained 23.9% of the total variance (Supplementary Table S1).

**Figure 1:**
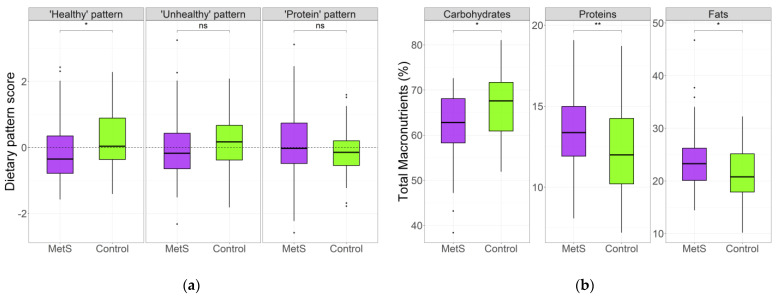
Dietary patterns and total macronutrient intake. (**a**) “Healthy”, “Unhealthy”, and “Protein” patterns adherence score in the study groups. (**b**) Percentage of the energy intake of total carbohydrates, proteins, and fats between the MetS women and controls. The plotted data represent medians and interquartile ranges. MetS: metabolic syndrome. ** *p* < 0.005; * *p* < 0.05; and ns, not significant.

The MetS group had significantly less adherence to the “healthy” pattern than the control group. No statistically significant differences were observed for “unhealthy” or “protein” pattern scores (Figure 1a). Regarding total macronutrient intake, significant differences between the women with MetS and the controls were observed; the median percentage of dietary carbohydrates in women with MetS was lower, while the median percentage of dietary protein and fat was higher than that in the control group (Figure 1b). These observations were particularly evident in the older women, who had significant differences in carbohydrate and fat proportions between age groups in the absence of MetS (Supplementary Figure S1). In addition, after adjusting for age, T2D diagnosis, as well as glucose-, lipid-, and blood pressure-lowering treatments, decreased carbohydrate intake and increased protein and fat intake remained significant in the MetS group (Supplementary Table S2).

### 3.3. Dietary Patterns Associated with MetS Risk Indicators

**Figure 2:**
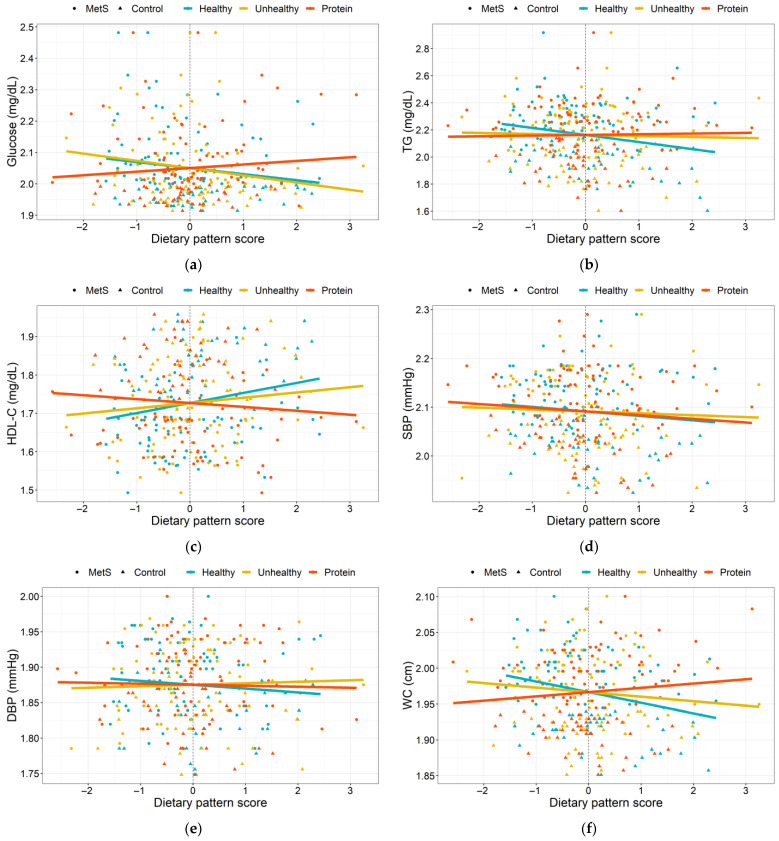
Dietary pattern adherence and MetS risk indicators. “Healthy”, “Unhealthy”, and “Protein” pattern score associations with (**a**) fasting glucose levels, (**b**) TG serum levels, (**c**) HDL-C serum levels, (**d**) systolic BP, (**e**) diastolic BP, and (**f**) waist circumference. The risk indicators values were log10 transformed. Associations were adjusted by age, T2D diagnosis, as well as glucose-, lipid-, and blood pressure-lowering treatments. MetS: metabolic syndrome; TG: triglycerides; HDL-C: high-density lipoprotein cholesterol; SBP: systolic blood pressure; DBP: diastolic blood pressure; BP: blood pressure; and WC: waist circumference.

Associations between the dietary pattern adherence and MetS risk indicators were evaluated in the entire study population (Figure 2). Interestingly, only the “healthy” pattern was positively associated with HDL-C levels (β = 0.044; *p* = 0.062), as well as negatively associated with TG concentration (β = −0.091; *p* = 0.051) and waist circumference (β = −0.028; *p* = 0.006); this last one remained significant after controlling for age, T2D diagnosis, as well as the use of glucose-, lipid-, and blood pressure-lowering drugs (Figure 2b,c,f; Supplementary Table S3). In addition, protein and fat intake percentage was positively correlated with fasting glucose levels and waist circumference, while carbohydrates were negatively correlated with these risk indicators. However, there were no statistical differences in total energy intake and physical activity between the study groups as these two traits correlated with the lipid profile and systolic blood pressure (Supplementary Figure S2).

### 3.4. Gut Microbiota Diversity and Taxonomic Composition of the MetS Women and Controls

There were statistical differences in the alpha diversity for the Shannon and Simpson indices, which were lower in the MetS group (Supplementary Figure S3A). Regarding beta diversity, a PERMANOVA analysis on the weighted UniFrac distance showed significant differences between the gut microbiota composition of the women with MetS and the controls (Supplementary Figure S3B,C). All of the estimators of alpha diversity were similar between the age groups (Supplementary Figure S4). 

The most abundant phylum in both study groups was Firmicutes (67.4% in the MetS group and 67.1% in the controls), followed by Bacteroidetes (18.8% and 22.6% in the MetS and control groups, respectively). The predominant classes were Clostridia (55.5% of the MetS group and 59.7% of the controls) and Bacteroidia (18.8% of the MetS group and 22.6% of the controls). Regarding genera, *Bacteroides* (10.3% and 13.7% in the MetS and control groups, respectively) and *Blautia* (11.0% and 11.7%, respectively) were the most abundant in the women with MetS and the controls (Supplementary Figure S5A–C). In addition, with LEfSe analysis, five genera were identified as enriched in the MetS group, whereas 14 genera were significantly higher in the controls (Supplementary Figure S6). Similarly, an LEfSe analysis of the older women and those younger than 65 years was consistent with most of the enriched taxa previously identified in the study groups. Additionally, the genera *Allisonella* and *DTUO14*, as well as *RF39* and *Rikenella*, were enriched in young women in the MetS and control groups, respectively, while *Alistipes*, *Alloprevotella*, and *Bacteroides* were enriched in the older control women (Supplementary Figure S7A–C).

**Figure 3:**
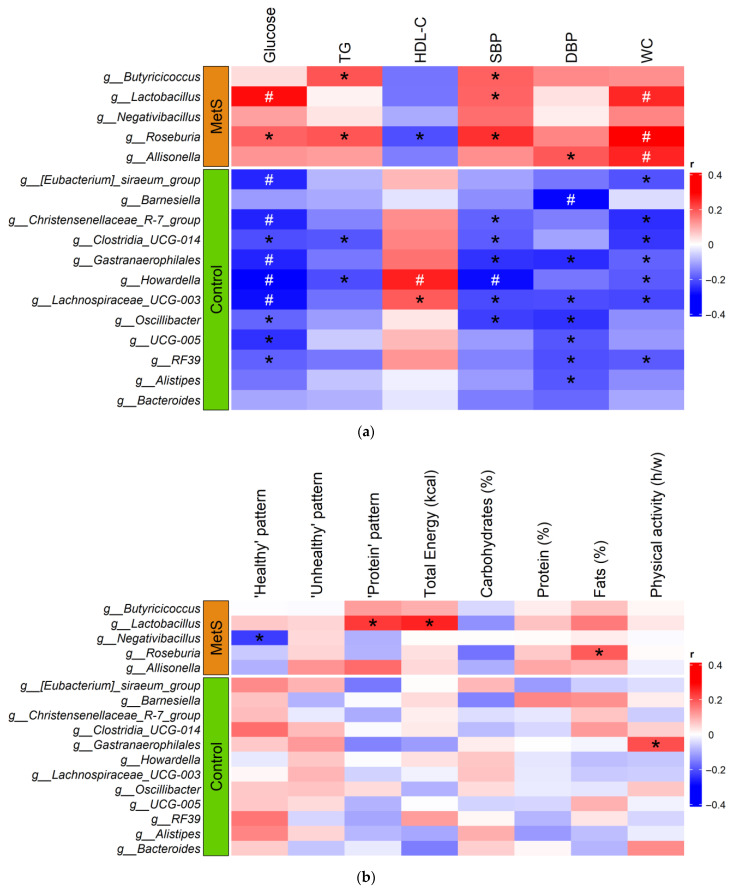
Heatmap of the Spearman correlations of relative genera abundance with (**a**) MetS risk indicators, and (**b**) nutritional traits and macronutrients. Red squares indicate positive correlations and blue squares indicate negative correlations. TG: triglycerides; HDL-C: high-cholesterol lipoprotein cholesterol; SBP: systolic blood pressure; DBP: diastolic blood pressure; WC: waist circumference; and MetS, metabolic syndrome. ^#^ *q* < 0.05 and * *p* < 0.05.

A total of 17 of the 27 differentiated genera, found in at least 10% of the population and with a mean abundance higher than 1%, were correlated with MetS risk indicators and nutrient traits in the whole population. As expected, MetS-enriched genera positively correlated with glucose, TG, blood pressure, and waist circumference, as well as negatively correlated with HDL-C, whereas the genera that were more abundant in the controls correlated in the opposite direction with the risk factors. Remarkably, *Lactobacillus*, *Roseburia*, *Allisonella*, *[Eubacterium] siraeum group*, *Barnesiella*, *Christensenellaceae R-7 group*, *Gastranaerophilales*, *Howerdella*, and *Lachnospiraceae UCG-003* correlations remained significant after correcting for multiple tests (*q* < 0.05; Figure 3a; Supplementary Table S6).

### 3.5. Influence of Diet on Gut Microbiota Composition

Alpha diversity indices were not associated with adherence to the dietary patterns, even though the gut microbiota composition showed significant differences regarding the weighted UniFrac distance and the “unhealthy” pattern (R^2^ = 0.016; F-value = 2.110; *p*-value = 0.011; Supplementary Figure S8C; Supplementary Tables S4 and S5). Furthermore, three of the genera enriched in the MetS group (*Lactobacillus*, *Negativibacillus*, and *Roseburia*) were significantly correlated with the “healthy” and “protein” dietary patterns, total energy intake, and percentage of fats, whereas only one genus (*Gastranaerophilales*) that had an increased abundance in the controls was positively correlated with physical activity (Figure 3b; Supplementary Table S7). Remarkably, *Roseburia*, *Negativibacillus*, and *Gastranaerophilales* associations remained significant after adjusting for age, T2D diagnosis, as well as glucose, lipid, and blood pressure-lowering treatments (Supplementary Table S8).

### 3.6. Roseburia Abundance Mediation Effect in Dietary Intake and MetS Risk Indicators

**Figure 4:**
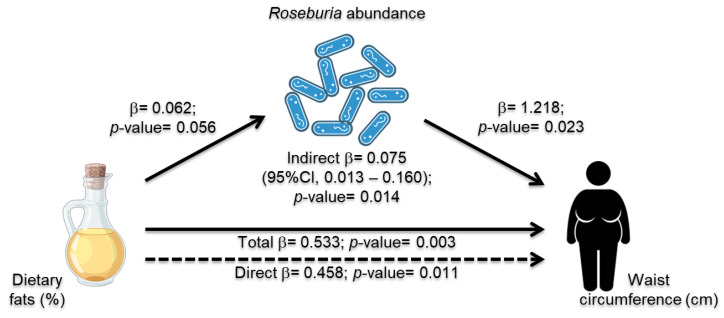
Relationship between the fat intake, waist circumference, and mediation effect of *Roseburia* abundance.

As there were consistent significant associations between the waist circumference–MetS risk indicators, fat intake, and abundance of *Roseburia*, the mediation analysis for these variables was conducted for the entire sample. Dietary fat was positively associated with waist circumference, which is consistent with previous analysis. *Roseburia* abundance, which was also positively associated with these nutritional traits, could mediate 7.5% of the relationship (Figure 4). Even though these associations remained significant after adjusting for age, a significantly greater abundance of *Roseburia* was observed in the younger women with MetS than in the controls from the same age group (<65 years) (who also reported a greater dietary fat intake) (Supplementary Table S9 and Figure S9A). Moreover, there were no statistically significant differences between the three variables in the mediation analysis according to the presence of T2D (Supplementary Figure S9C–E).

## 4. Discussion

Consistently across different populations, a greater adherence to a prudent dietary pattern has been associated with lower MetS risk [12,22]. In this regard, aligning with prudent dietary patterns characterized as nutrient-dense and high in vegetables, fruits, and whole grains reduces the risk of MetS and its related metabolic disorders [36]. 

This study identified three similar dietary patterns to those previously reported in the “The Health Workers Cohort Study”; however, only the “healthy” pattern presented a significantly lower adherence in women with MetS. This finding suggests that, compared to the whole population that did not show an association with the prudent pattern, Mexican postmenopausal women would have different dietary habits than men or younger women (<45 years old); therefore, dietary requirements should be assessed according to sex and age to better understand metabolic-related alterations.

Furthermore, in the present study, a greater adherence to the “healthy” pattern was associated with lower waist circumference, lower TG levels, and increased HDL-C levels. Current evidence shows that weight gain and fat mass increase are related to dietary habits and sedentariness rather than exclusively menopause transition [37]. Moreover, visceral fat gain may interact with hormonal changes and dietary habits during this stage, thereby promoting a dysregulation of lipid metabolism [38]. Thus, maintaining a healthy diet after menopause may protect against the gain of central adiposity and dyslipidemia [39,40].

In addition to dietary patterns, total macronutrients in this study showed significant differences between the study groups, even though total energy intake was similar. Nevertheless, the percentage of these macronutrients was outside the daily recommendations for a healthy diet in both groups. The 2015–2020 Dietary Guideline for Americans recommends an intake of 45–65% carbohydrates, 25–35% fats, and 10–30% protein of the total calories [41]; however, we observed a higher consumption of carbohydrates together with a decreased ingestion of proteins in both groups. Notably, previous studies have reported an association between higher carbohydrate/lower protein intake and a higher risk of MetS, particularly in terms of abdominal obesity, in older Asian women [42,43]. Nonetheless, our study observed a lower carbohydrate/higher protein intake in women with MetS. These contradictory results may be related to the population evaluated and the methodology used to assess food consumption [43].

Recently, Peter et al. reported that the gut microbiota in Hispanic/Latino women contributes to changes in MetS risk indicators during menopause [11]. In conjunction with gut microbiota, the associations between menopause and metabolic traits are partly mediated by diet in postmenopausal women [9]. The present study observed decreased alpha diversity in Mexican postmenopausal MetS women, which is consistent with previous reports [44]. Furthermore, beta diversity differences were also significantly associated with an “unhealthy” dietary pattern, thus suggesting that a greater adherence to this diet might be unfavorable in shaping microbiota diversity. Human studies and rodent models have shown that the chronic consumption of a Westernized or a high-fat diet (HFD) alters gut microbiota. Furthermore, both contribute to intestinal barrier alterations that promote the passage of intestinal luminal content, thus leading to low-grade inflammation and the impairing of normal metabolic functions [45]. On the other hand, an ovariectomized mouse model revealed that the gut microbiota of menopausal obesity and HFD-induced obesity were very similar, even though some bacterial taxa were found when the two traits were combined [46]. Therefore, an inadequate diet in postmenopausal women could enhance gut microbiota alterations due to menopause status, and that these, in turn, could influence an increased risk of developing MetS.

Previous reports have been inconsistent with *Roseburia* enrichment or depletion in postmenopausal women. Interestingly, in a study that evaluated lean postmenopausal women, the abundance of *Roseburia* was decreased [47]. In contrast, another study that reported an increase in *Roseburia* in postmenopausal women was also characterized by the fact that these women presented obesity [48]. Moreover, in line with our findings, *Roseburia* has been reported to be enriched in obese Mexican women and those with MetS [45]. The genus *Roseburia*, which includes five species, consists of obligate Gram-positive anaerobic commensal bacteria that produce short-chain fatty acids (SCFAs), and they have been associated with anti-inflammatory effects and energy homeostasis [49]. An adherence to the prudent Mediterranean diet, which includes plant-based foods, whole grains, legumes, and nuts, has been associated with higher *Roseburia* abundance [50]. However, Schneeberger et al. reported that, in a diet-induced obese mouse model, an extended administration of 45% kcal (7:1 lard/soybean oil) HFD increased the abundance of *Roseburia* in the gut in contrast to a shorter period of HFD feeding [51,52]. In the present study, a higher fat intake was observed in women with MetS, and it was also positively correlated with *Roseburia* abundance and waist circumference. Notably, the mediation analysis revealed that *Roseburia* could explain 7% of the relationship between dietary fat and abdominal obesity when evaluated by waist circumference. Additionally, *Roseburia* can produce conjugated-linoleic acids (CLAs) from linoleic acid; thus, an increase in its abundance could be related to HFD by supplying substrates to this bacterium [53]. However, saturated and unsaturated dietary fat supplementation studies and gene expression evaluation are required to understand the *Roseburia* role in dietary fat metabolism. 

*Lactobacillus* is a widely studied genus, with some species used as probiotics to alleviate inflammation and improve metabolic syndrome-related traits; however, in this study, the abundance of *Lactobacillus* was increased in women with MetS. Moreover, this genus was positively correlated with an adherence to the “protein” dietary pattern. These findings coincide with those of a short-term study in which *Lactobacillus* abundance increased in meat-fed rats compared to those on a vegetal protein diet; however, the underlying mechanisms of this require further research [54]. 

*Negativibacillus* is a recently identified harmful genus of human gut microbiota that has been reported to be increased in an obesity-associated metabolic syndrome mice model [55]. Furthermore, the relative abundance of *Negativibacillus* was significantly enriched in 60% HFD-fed mice, and this was positively associated with a higher consumption of ultra-processed foods in obese women and men with MetS [56,57]. Consistent with those previous reports, the abundance of *Negativibacillus* was higher in MetS women and was also negatively correlated with an adherence to the “healthy” dietary pattern in this study. However, as there is little information in the literature on the characteristics of this bacterium, additional studies are required to establish its participation in metabolic dysregulation and its relationship with diet.

In contrast, the genera enriched in the control group included many known SCFA-producing bacteria that are negatively associated with adverse metabolic traits, such as MetS. In addition, some studies in human and animal models have reported that HFD decreases fecal and the blood amounts of SCFA, and this is probably due to alterations in the microbiota composition and diet-induced obesity [58]. Furthermore, recently, Pessoa et al. showed that, besides SCFA bacteria, HFD decreased the abundance of *Gastranaerophilales* more than a high-sugar diet [59]. This bacterium of an unknown role in the human gut microbiota was also found to be enriched in the controls in this study and positively correlated with physical activity, and its beneficial effects could also be related to the exercise of adjuvant actions.

Finally, although this study was performed on a specific population group that was well selected, one of its limitations include that it was a cross-sectional sampling. This is an issue as longitudinal studies will be required to determine whether dietary patterns influence menopause–gut microbiota changes and affect women’s metabolism during this stage. Moreover, a further evaluation of dietary macronutrient subclasses, micronutrients, and circulating sex hormone levels is required to establish the impact of dietary patterns on the microbiome composition during menopause.

## 5. Conclusions

The development of metabolic syndrome in postmenopausal women is a complex process that involves a combination of factors including hormonal changes, microbiota composition, and dietary patterns. Notably, at this stage of a women’s life, gut microbiota changes could be influenced by particular dietary habits, which is different from other study groups. Interventions that target these factors may help prevent or reduce the risk of metabolic syndrome in postmenopausal women. Additional studies will be needed to identify other health implications of menopause–gut microbiome interactions. Furthermore, identifying favorable dietary patterns that influence the gut microbiota could contribute to improving women’s health at this stage of life.

## Figures and Tables

**Table 1:** Characteristics of the study groups.

	MetS (*n* = 68)	Control (*n* = 48)	*p*-Value
Age, years	67.5 (58.0–73.5)	57.5 (52.3–63.0)	1.0 × 10^−4^
≥65 years old, *n* (%)	40 (58.8)	11 (22.9)	1.2 × 10^−4^
BMI, kg/m^2^	30.6 (28.2–34.5)	23.6 (21.9–24.7)	5.0 × 10^−18^
Glucose, mg/dL	115.5 (104.0–144.5)	91.0 (86.3–96.0)	4.6 × 10^−19^
TG, mg/dL	188.0 (162.5–237.3)	99.5 (72.8–121.3)	2.9 × 10^−16^
HDL-C, mg/dL	46.1 (39.8–49.4)	67.9 (57.7–76.9)	2.2 × 10^−15^
SBP, mmHg	139.0 (125.0–148.8)	106.0 (98.5–114.8)	1.0 × 10^−16^
DBP, mmHg	79.5 (70.3–86.8)	69.5 (64.3–75.0)	9.4 × 10^−8^
WC, cm	100.0 (95.0–106.0)	82.5 (79.3–85.0)	5.5 × 10^−20^
T2D, *n* (%)	44 (64.7)	0 (0)	1.5 × 10^−12^
Hypoglycemic treatment, *n* (%)	33 (48.5)	0 (0)	1.2 × 10^−8^
Hypolipidemic treatment, *n* (%)	32 (47.1)	0 (0)	2.3 × 10^−8^
Antihypertensive treatment, *n* (%)	33 (48.5)	0 (0)	1.2 × 10^−8^
Total energy intake, kcal/day	1737.6 (1065.4–2415.4)	1475.7 (1218.9–1830.1)	0.223
Physical activity, hours per week	0.76 (0.16–3.50)	2.04 (0.47–4.76)	0.061

Data are presented as medians (interquartile ranges) or numbers (percentages). MetS: metabolic syndrome; BMI: body mass index; TG: triglycerides; HDL-C: high-density lipoprotein cholesterol; SBP: systolic blood pressure; DBP: diastolic blood pressure; WC: waist circumference; and T2D: type 2 diabetes.

## Data Availability

The data supporting the findings of this study are available from the corresponding author (R.V.-C.) upon reasonable request.

## Supplementary Materials

The following supporting information can be downloaded at: https://www.mdpi.com/article/10.3390/nu15224704/s1, Table S1: Factor-loading matrix for dietary patterns in postmenopausal women; Table S2: Association of dietary total macronutrients with MetS; Table S3: Association of dietary patterns scores with metabolic syndrome risk indicators; Table S4: Association of dietary pattern scores with gut microbiota alpha diversity estimators; Table S5: Pairwise PERMANOVA of the gut microbiota beta diversity based on UniFrac distances and dietary patterns; Table S6: Spearman correlations of the relative genera abundance with MetS risk indicators; Table S7: Spearman correlations of the relative genus abundance with nutritional traits and macronutrients; Table S8: Associations between the microbiota abundance, MetS risk indicators, and nutritional traits; Table S9: Mediation analysis of the *Roseburia* abundance with fat intake and waist circumference; Figure S1: Percentage of the total macronutrient intake between age groups; Figure S2: Heatmap of the Spearman correlations of nutritional traits and macronutrients with MetS risk indicators; Figure S3: Gut microbiota diversity indicators in the MetS group and control group; Figure S4: Alpha diversity parameters between the age groups; Figure S5: Relative abundance distribution of the gut microbiota in women with MetS and the controls; Figure S6: Differences in the taxonomic gut microbiota, as identified by LEfSe analysis, between women with MetS and the controls; Figure S7: Taxonomic gut microbiota differences, as identified by LEfSe analysis, between the women with MetS and the controls; Figure S8: Principal coordinate analysis of the weighted and unweighted UniFrac distances for dietary pattern tertiles; and Figure S9: Mediation analysis of the variable levels according to the cofounders.
