# Supplementary material for: Dietary Patterns Are Associated with the Gut Microbiome and Metabolic Syndrome in Mexican Postmenopausal Women
Source: Nutrients. 2023 Nov 7;15(22):4704. doi: 10.3390/nu15224704 (PMC10675332; doi:10.3390/nu15224704)
Supplement: Supplementary file 1 [file nutrients-15-04704-s001.zip › supplementals 1.pdf]

Supplementary Table S1. Factor-loading matrix for dietary patterns in postmenopausal women.

	Dietary patterns[1]		
	**Healthy**	**Unhealthy**	**Protein**
**Variance explained (%)**	8.5	8.0	7.4
Corn tortilla	-0.4396	-	-
Mexican food	-	-	-
Whole grains	-	-	-
Refined grains	-0.3431	-	-
Pastry	-	0.3133	-0.5073
Desserts	-0.3040	-	-0.4661
Snacks	-	-	-
Fresh vegetables	0.4608	-0.3502	-
Tomato juice	-	-	-
Potatoes	-	-	0.3794
Fresh fruit	0.5323	-	-
Fruit juices	0.4209	-	-
Eggs	-	-	0.3571
Poultry	-	-	0.3687
Red meat	-	-	0.3613
Processed meat	-	-	0.5852
Fish and seafood	0.3945	-	0.3102
Low-fat dairy products	-	-	-
High-fat dairy products	-	-	-
Legumes	-	-0.5893	-
Oils and nuts	0.6739	-	-
Butter	-0.3790	-	0.3606
Sweets and sugar	-	0.5707	-
Sodas	-	0.4192	-
Other sweetened beverages	-0.3154	-0.3019	-0.3345
Low-energy drinks	-	0.4626	-
Alcohol	0.3156	0.3947	-
Tea and caffeine	-	0.5635	-
Water	-	-	0.3947

[1]Values <0.3 were excluded for simplicity.

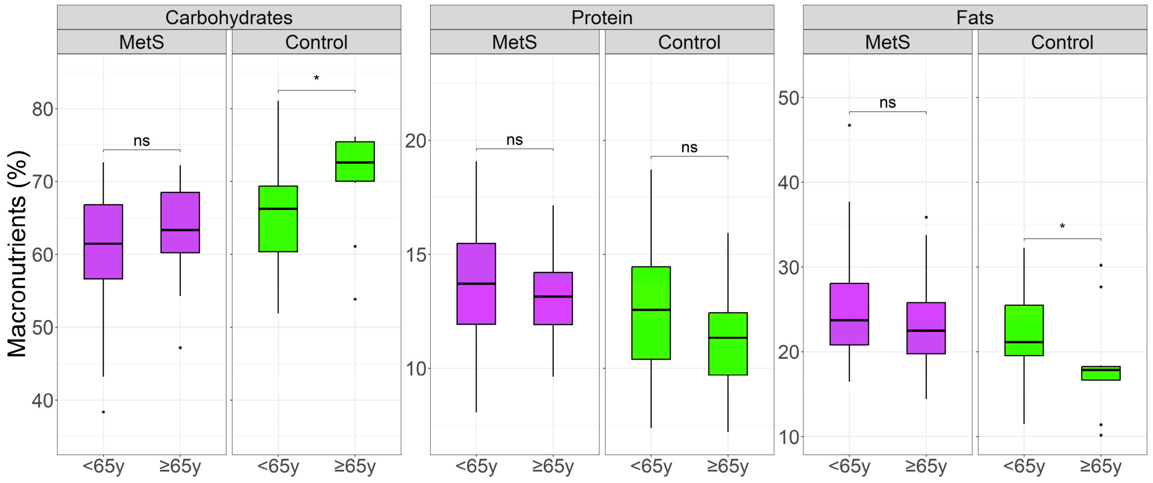

Supplementary Figure S1. Percentage of total macronutrient intake between age groups (<65 years n=65; ≥65 years n=51). The plotted data represent medians and interquartile ranges. MetS, Metabolic syndrome. *p < 0.05; ns, not significant.

Supplementary Table S2. Association of dietary total macronutrients with MetS.

	**Unadjusted**			**Adjusted for age, T2D, and glucose-, lipid- and blood pressure-lowering treatment**		
	**Coefficient**	***p*-value**	***q*-value**	**Coefficient**	***p*-value**	***q*-value**
Carbohydrates	-0.472	2.1×10^{-19}	**1.2×10^{-18}**	-0.472	3.3×10^{-14}	**6.5×10^{-13}**
Fat	0.212	0.012	**0.024**	0.218	0.051	0.132
Protein	0.209	0.003	**0.007**	0.235	0.014	0.058

Coefficients from the generalized linear model using MaAsLin2 on pairwise testing between the MetS and control groups. *q*-values were calculated using FDR correction. MetS: Metabolic Syndrome.

Supplementary Table S3. Association of dietary patterns scores with Metabolic Syndrome risk indicators.

Linear Regression		Healthy pattern			Unhealthy pattern			Protein pattern		
		**Beta**	**95% CI**	***p*-value**	**Beta**	**95% CI**	***p*-value**	**Beta**	**95% CI**	***p*-value**
Glucose	Model I[1]	-0.044	-0.096, 0.007	0.092	-0.053	-0.109, 0.003	0.061	0.026	-0.028, 0.080	0.337
	Model II[2]	-0.008	-0.043, 0.026	0.624	-0.018	-0.056, 0.019	0.338	0.013	-0.025, 0.050	0.508
TG	Model I[1]	-0.120	-0.217, -0.023	**0.016**	-0.017	-0.125, 0.091	0.754	0.012	-0.091, 0.115	0.823
	Model II[2]	-0.091	-0.183, 0.0004	0.051	0.031	-0.070, 0.132	0.548	0.001	-0.099, 0.102	0.978
HDL-C	Model I[1]	0.060	0.011, 0.110	**0.018**	0.032	-0.023, 0.086	0.254	-0.023	-0.076, 0.029	0.379
	Model II[2]	0.044	-0.002, 0.090	0.062	0.009	-0.041, 0.060	0.711	-0.021	-0.072, 0.029	0.409
SBP	Model I[1]	-0.021	-0.055, 0.014	0.233	-0.009	-0.046, 0.029	0.637	-0.017	-0.053, 0.018	0.336
	Model II[2]	-0.018	-0.046, 0.010	0.212	0.017	-0.013, 0.048	0.260	-0.004	-0.034, 0.027	0.804
DBP	Model I[1]	-0.012	-0.037, 0.012	0.315	0.005	-0.022, 0.031	0.718	-0.003	-0.029, 0.022	0.800
	Model II[2]	-0.005	-0.029, 0.019	0.665	0.010	-0.016, 0.035	0.456	-0.010	-0.035, 0.016	0.448
WC	Model I[1]	-0.034	-0.058, -0.011	**0.005**	-0.015	-0.041, 0.012	0.273	0.014	-0.011, 0.039	0.278
	Model II[2]	-0.028	-0.048, -0.008	**0.006**	-0.0001	-0.022, 0.022	0.993	0.008	-0.014, 0.030	0.468

Risk indicators values were log10 transformed. TG, Triglycerides; HDL-C, High-density lipoprotein cholesterol; SBP, Systolic blood pressure; DBP, Diastolic blood pressure; WC, Waist circumference.

[1]Non-adjusted linear regression model.

[2]Model I adjusted by age, T2D diagnosis, and the use of glucose-, lipid- and blood pressure-lowering drugs.

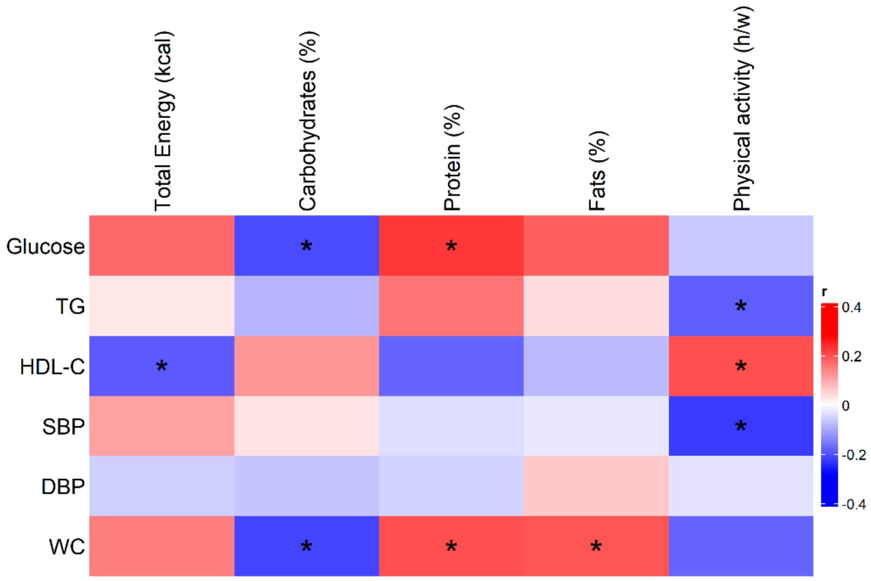

Supplementary Figure S2. Heatmap of Spearman correlations of nutritional traits and macronutrients with MetS risk indicators (n=116). Red squares indicate positive correlations, and blue squares indicate negative correlations. TG, Triglycerides; HDL-C, High-density lipoprotein cholesterol; SBP, Systolic blood pressure; DBP, Diastolic blood pressure; WC, Waist circumference. *$p < 0.05$.

A.

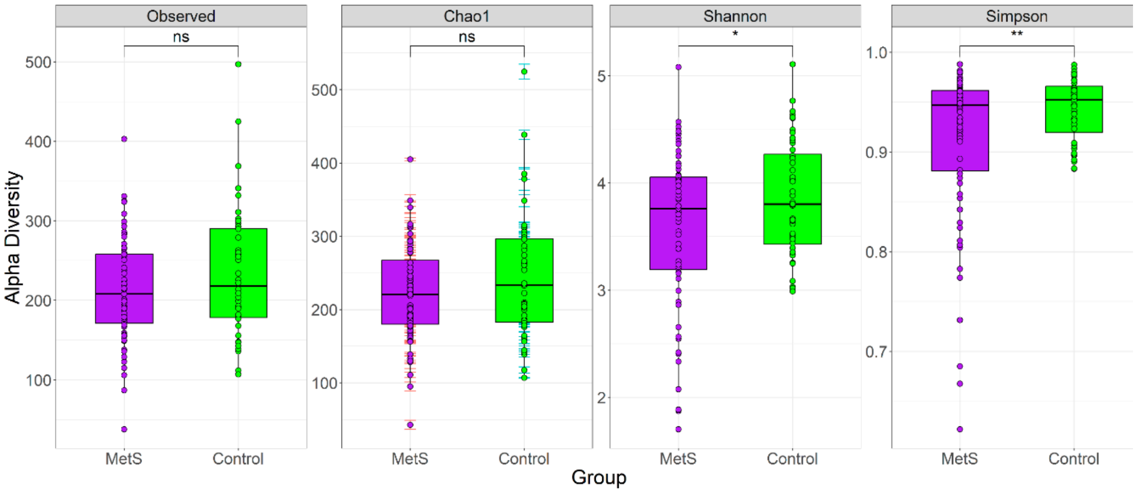

B.

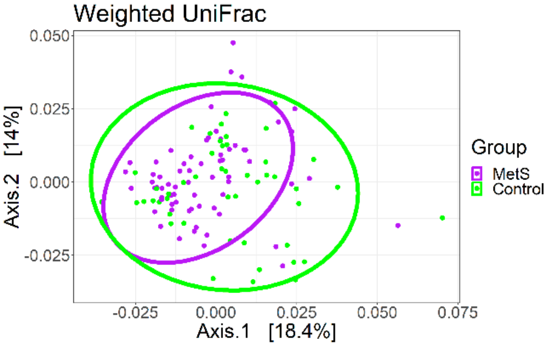

C.

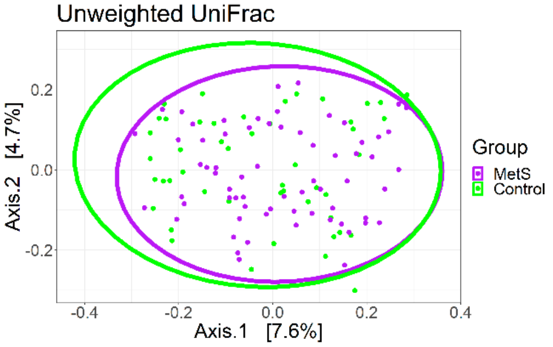

R^2 = 0.016; F-value = 2.110; *p*-value = 0.011

R^2 = 0.010; F-value = 1.150; *p*-value = 0.117

Supplementary Figure S3. Gut microbiota diversity indicators in MetS group (n=68) and controls (n=48). (A) Alpha diversity parameters; the plotted data represent medians and interquartile ranges. (B-C) Principal Coordinate Analysis of the Weighted and Unweighted UniFrac distances, respectively; points are colored by study group; differences were assessed with PERMANOVA analysis adjusted by age, T2D diagnosis, use of glucose-, lipid- and blood pressure-lowering drugs, and random technical covariates (extraction kit and sequencing pool). MetS, Metabolic syndrome. **$p < 0.005$; *$p < 0.05$; ns, not significant.

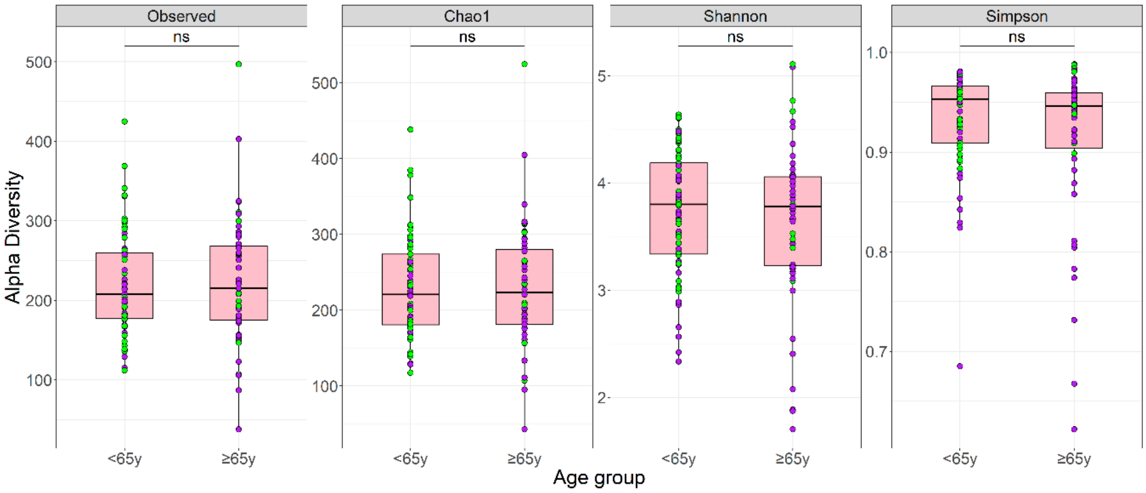

Supplementary Figure S4. Alpha diversity parameters between age groups (<65 years n=65; ≥65 years n=51); the plotted data represent medians and interquartile ranges; points are colored by study group, MetS: purple; Controls: green. MetS, Metabolic syndrome. ns, not significant.

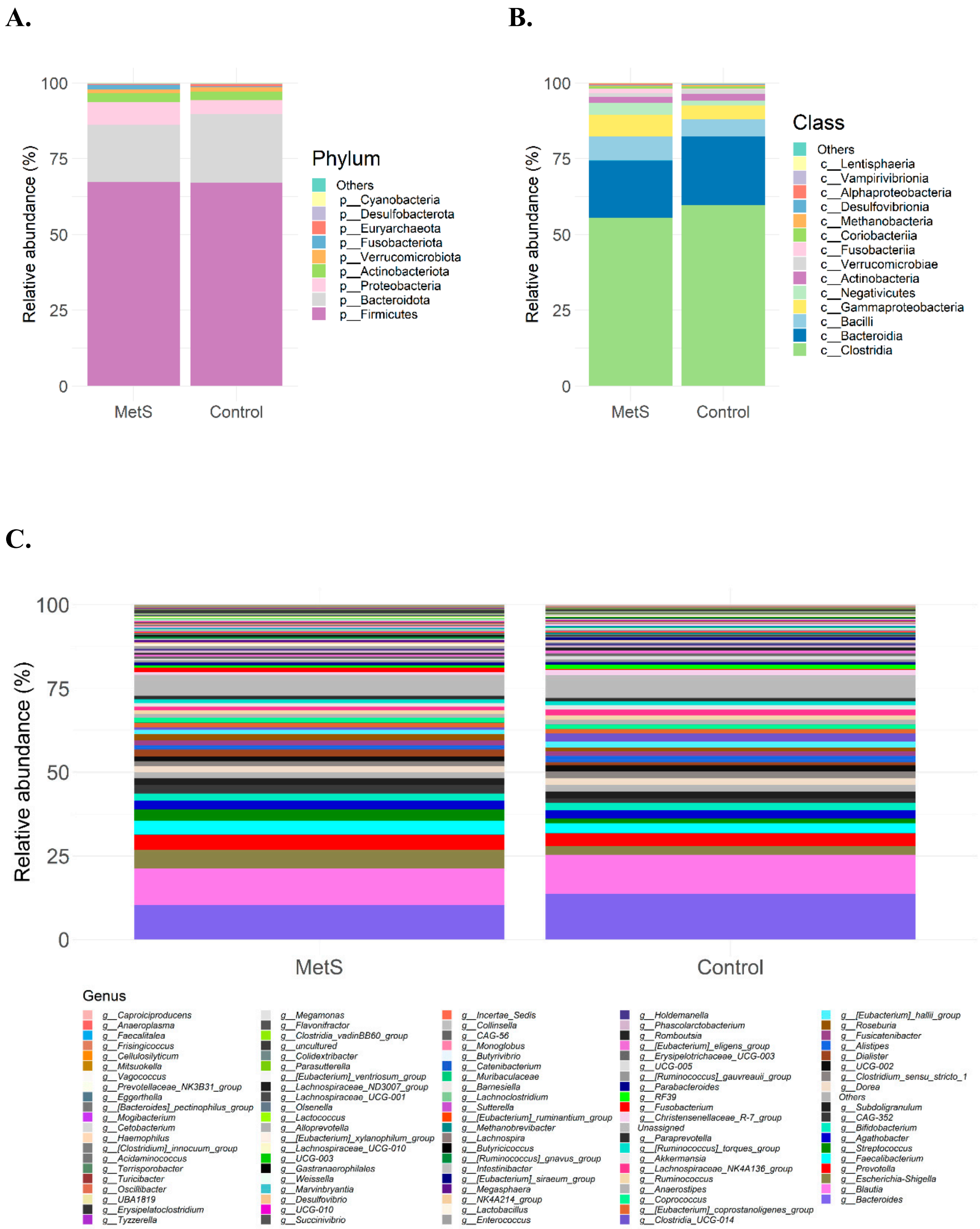

Supplementary Figure S5. Relative abundance distribution of gut microbiota in MetS women and controls. Bacterial composition at (A) Phylum level, (B) Class level, (C) Genus level.

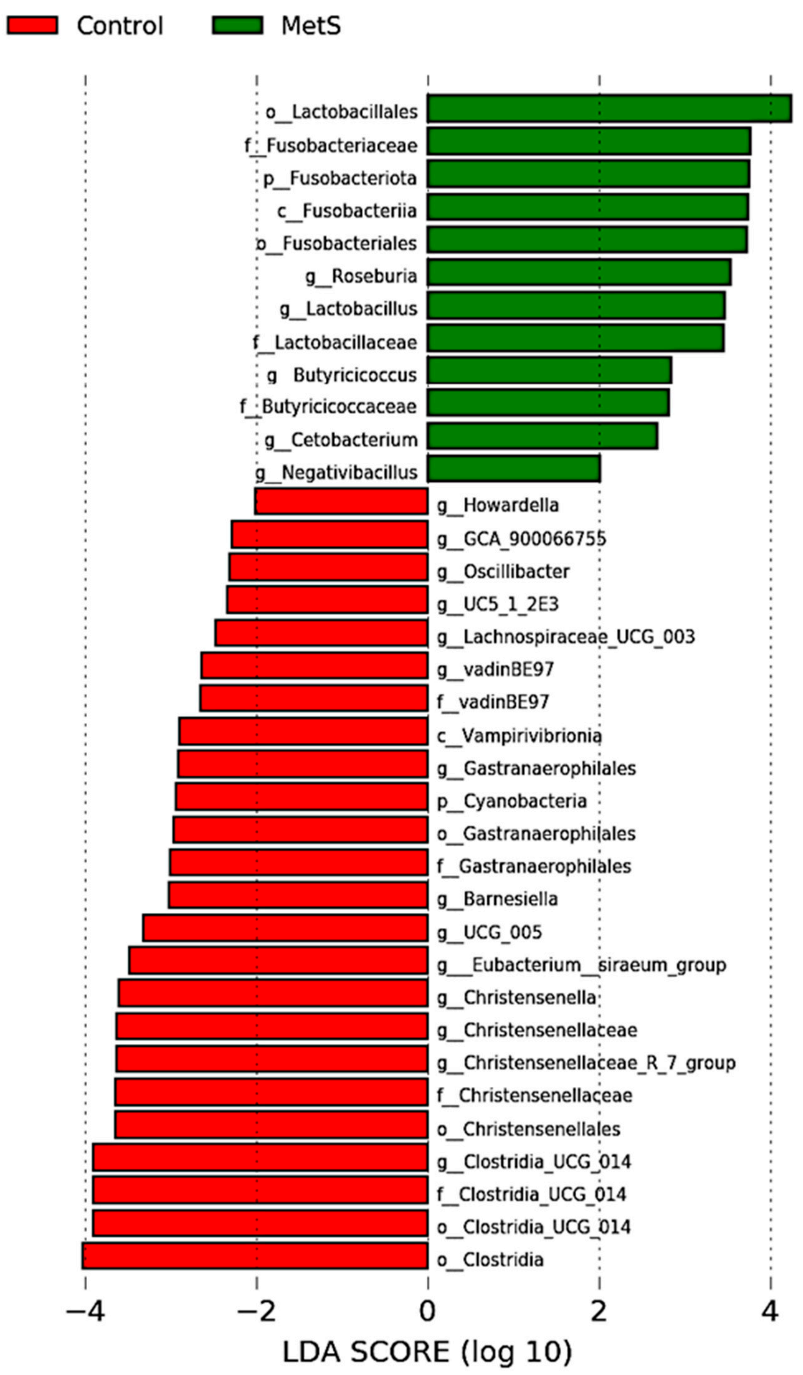

Supplementary Figure S6. Taxonomic gut microbiota differences identified by LEfSe analysis between MetS women (green) and controls (red). MetS, Metabolic syndrome; (p) phyla; (c) class; (o) order; (f) family; (g) genus. Taxa with LDA score > 2.0 and $p < 0.05$ are shown.

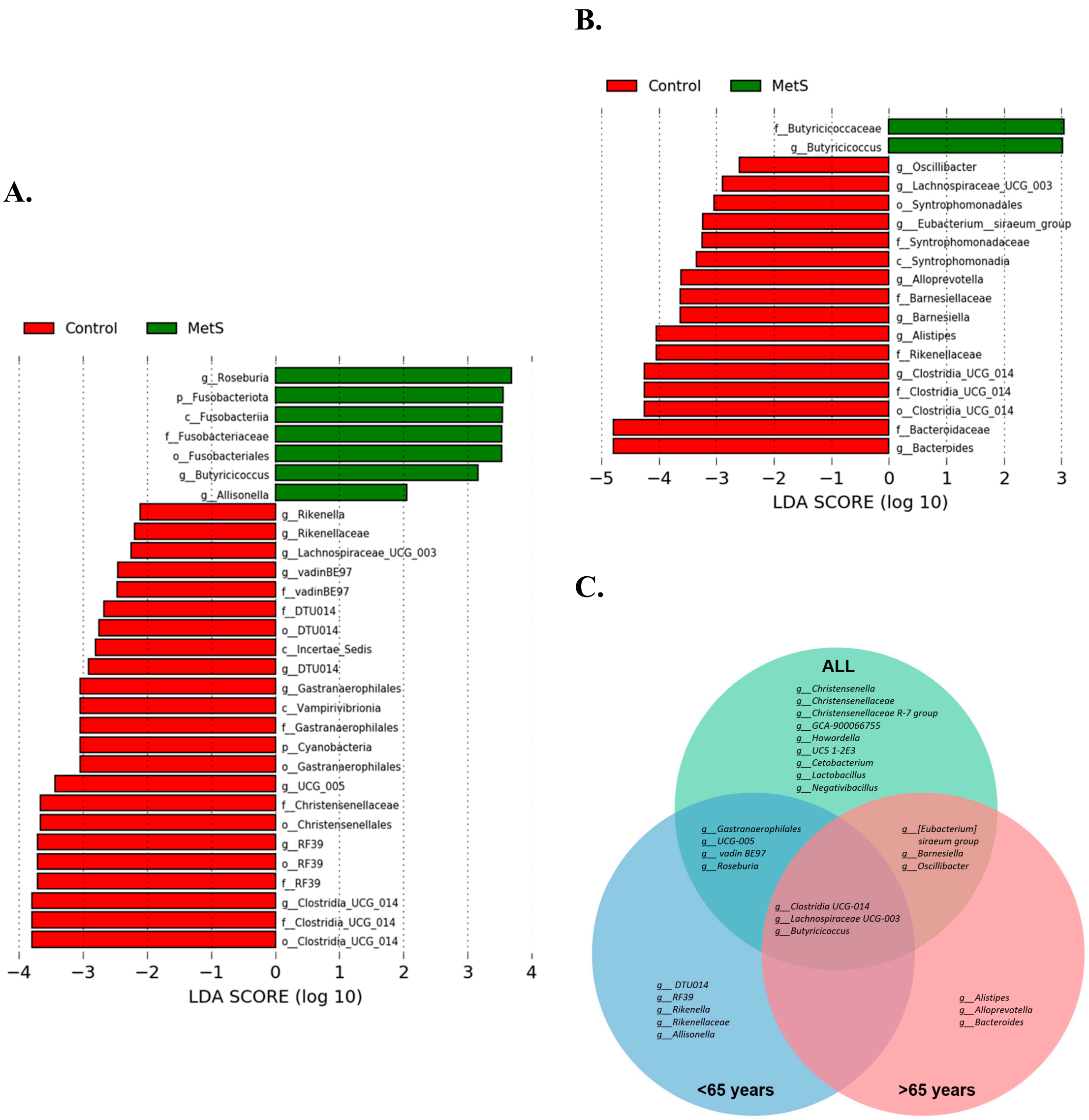

Supplementary Figure S7. Taxonomic gut microbiota differences identified by LEfSe analysis between MetS (green) and controls (red) of (A) younger and (B) older women. (C) Venn Diagram showing the overlap of enriched genera identified. MetS, Metabolic syndrome; (p) phyla; (c) class; (o) order; (f) family; (g) genus. Taxa with LDA score > 2.0 and $p < 0.05$ are shown.

A.

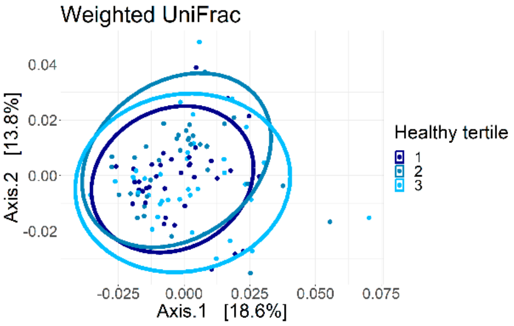

$R^2 = 0.015$; F-value = 0.898; *p*-value = 0.623

B.

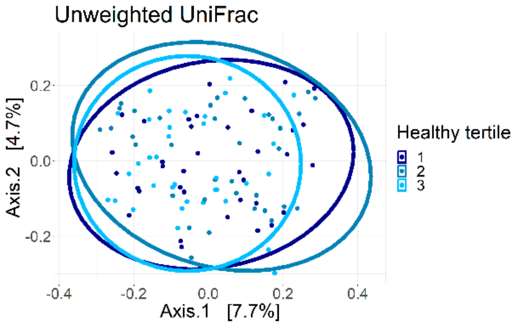

$R^2 = 0.021$; F-value = 1.155; *p*-value = 0.064

C.

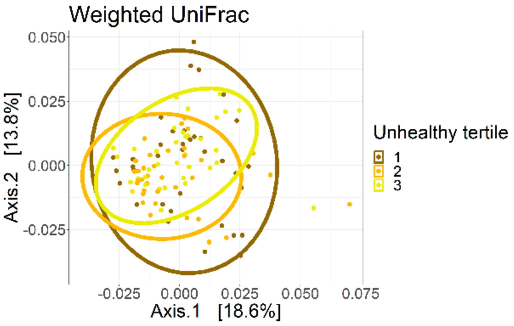

$R^2 = 0.025$; F-value = 1.551; *p*-value = 0.033

D.

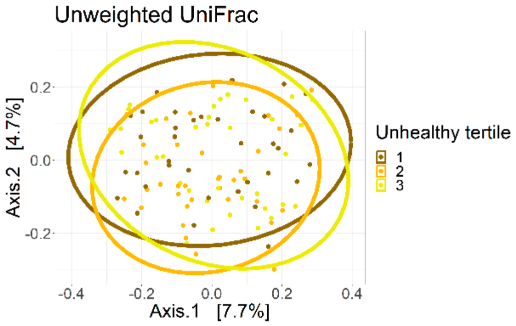

$R^2 = 0.019$; F-value = 1.033; *p*-value = 0.311

E.

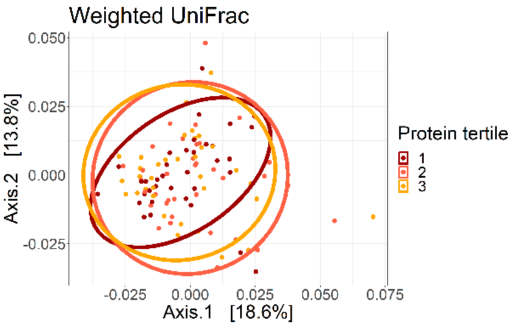

$R^2 = 0.016$; F-value = 1.016; *p*-value = 0.420

F.

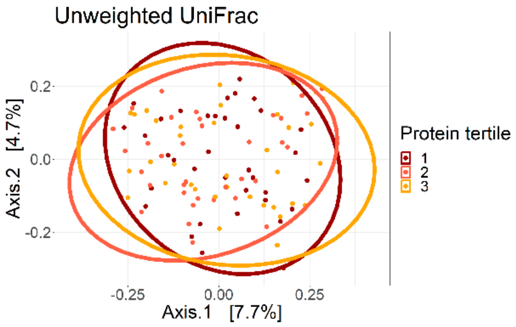

$R^2 = 0.018$; F-value = 1.016; *p*-value = 0.370

Supplementary Figure S8. Principal Coordinate Analysis of the Weighted and Unweighted UniFrac distances for dietary pattern tertiles, respectively. (A-B) “Healthy” pattern. (C-D) “Unhealthy” pattern. (E-F) “Protein” pattern. Points are colored by tertile grouping. Differences were assessed with PERMANOVA analysis adjusted by age, T2D diagnosis, use of glucose-, lipid- and blood pressure-lowering drugs, and random technical covariates (extraction kit and sequencing pool).

Supplementary Table S4. Association of dietary patterns scores with gut microbiota alpha diversity estimators.

**Linear Regression**		**Healthy pattern**			**Unhealthy pattern**			**Protein pattern**		
		**Beta**	**95% CI**	***p*-value**	**Beta**	**95% CI**	***p*-value**	**Beta**	**95% CI**	***p*-value**
Observed	Model I[1]	4.89	-9.197, 18.967	0.493	-0.31	-15.522, 14.911	0.968	-0.81	-15.378, 13.756	0.912
	Model II[2]	2.47	-11.203, 17.529	0.731	-2.20	-18.197, 13.322	0.778	3.27	-11.203, 17.529	0.686
Chao1	Model I[1]	5.04	-9.495, 19.577	0.493	0.35	-15.360, 16.053	0.965	0.75	-14.288, 15.786	0.922
	Model II[2]	2.75	-11.287, 18.150	0.709	-1.56	-17.694, 14.612	0.846	4.85	-12.694, 19.707	0.543
Shannon	Model I[1]	0.12	-0.012, 0.247	0.075	0.06	-0.085, 0.197	0.435	-0.06	-0.197, 0.073	0.365
	Model II[2]	0.10	-0.030, 0.224	0.134	0.03	-0.123, 0.160	0.650	-0.001	-0.145, 0.138	0.985
Simpson	Model I[1]	0.01	-0.0003, 0.026	0.055	0.008	-0.006, 0.023	0.248	-0.001	-0.016, 0.012	0.808
	Model II[2]	0.01	-0.0009, 0.025	0.077	0.005	-0.010, 0.018	0.462	0.004	-0.011, 0.017	0.591

[1]Non-adjusted linear regression model.
[2]Model I adjusted by age, T2D diagnosis, use of glucose-, lipid- and blood pressure-lowering drugs, and random technical covariates (extraction kit, and sequencing plate).

Supplementary Table S5. Pairwise PERMANOVA analysis of gut microbiota beta diversity by UniFrac distances and dietary patterns.

PERMANOVA		Weighted UniFrac			Unweighted UniFrac		
		R^2	F-value	*p*-value	R^2	F-value	*p*-value
MetS	Model I[1]	0.016	1.867	**0.028**	0.010	1.107	0.171
	Model II[2]		2.110	**0.011**		1.150	0.117
Healthy pattern	Model I[1]	0.015	0.786	0.807	0.021	1.111	0.120
	Model II[2]		0.898	0.623		1.155	0.064
Unhealthy pattern	Model I[1]	0.025	1.359	0.094	0.019	0.996	0.450
	Model II[2]		1.551	**0.033**		1.033	0.311
Protein pattern	Model I[1]	0.016	0.887	0.634	0.018	0.978	0.538
	Model II[2]		1.016	0.420		1.016	0.370

[1]Non-adjusted PERMANOVA model.
[2]Model I adjusted by age, T2D diagnosis, use of glucose-, lipid- and blood pressure-lowering drugs, and random technical covariates (extraction kit and sequencing pool).

Supplementary Table S6. Spearman correlations of relative genera abundance with MetS risk indicators. (Excel file)

Supplementary Table S7. Spearman correlations of relative genera abundance with nutritional traits and macronutrients. (Excel file)

Supplementary Table S8. Association of microbiota abundance with MetS risk indicators and nutritional traits. (Excel file)

Supplementary Table S9. Mediation analysis of Roseburia abundance with fats intake and waist circumference.

**Mediation analysis**		**Beta**	**95% CI**	***p*-value**
Fats vs *Roseburia*	Model I[1]	0.056	-0.007, 0.119	0.081
	Model II[2]	0.062	-0.002, 0.125	0.056
Roseburia vs WC	Model I[1]	1.452	0.346, 2.558	**0.011**
	Model II[2]	1.218	0.171, 2.265	**0.023**
Direct effect (Fats vs WC)	Model I[1]	0.338	-0.028, 0.703	0.070
	Model II[2]	0.458	0.108, 0.807	**0.011**
Total effect (Fats + *Roseburia* vs WC)	Model I[1]	0.419	0.049, 0.789	**0.027**
	Model II[2]	0.533	0.183, 0.884	**0.003**
Indirect effect (*Roseburia* mediation)	Model I[3]	0.081	0.018, 0.180	**0.010**
	Model II[3]	0.075	0.013, 0.160	**0.014**

WC, Waist circumference.
[1]Non-adjusted linear regression model.
[2]Model I adjusted by age and random technical covariates (extraction kit, and sequencing plate).
[3]Mediation effect analysis

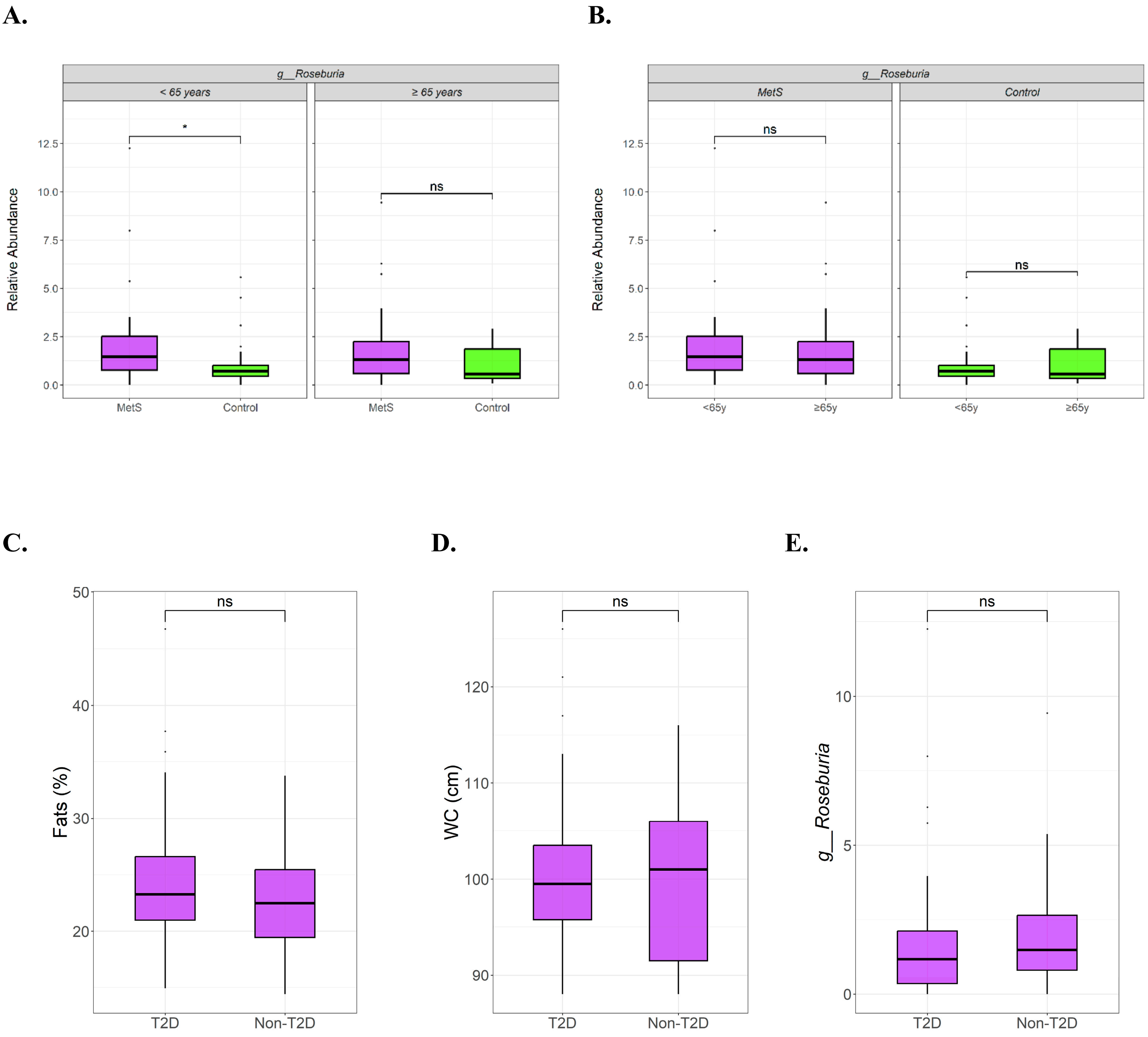

Supplementary Figure S9. Mediation analysis variables' levels according to cofounders. (A) Roseburia abundance between study groups according to age. (B) Roseburia abundance between age groups according to MetS classification. (C) Fat intake, (D) WC, and (E) Roseburia abundance between MetS women with and without T2D diagnosis. WC: waist circumference. *$p < 0.05$; ns, not significant.
